# WaveSeq: A Novel Data-Driven Method of Detecting Histone Modification Enrichments Using Wavelets

**DOI:** 10.1371/journal.pone.0045486

**Published:** 2012-09-28

**Authors:** Apratim Mitra, Jiuzhou Song

**Affiliations:** Department of Animal and Avian Sciences, University of Maryland, College Park, Maryland, United States of America; Georgia Institute of Technology, United States of America

## Abstract

**Background:**

Chromatin immunoprecipitation followed by next-generation sequencing is a genome-wide analysis technique that can be used to detect various epigenetic phenomena such as, transcription factor binding sites and histone modifications. Histone modification profiles can be either punctate or diffuse which makes it difficult to distinguish regions of enrichment from background noise. With the discovery of histone marks having a wide variety of enrichment patterns, there is an urgent need for analysis methods that are robust to various data characteristics and capable of detecting a broad range of enrichment patterns.

**Results:**

To address these challenges we propose WaveSeq, a novel data-driven method of detecting regions of significant enrichment in ChIP-Seq data. Our approach utilizes the wavelet transform, is free of distributional assumptions and is robust to diverse data characteristics such as low signal-to-noise ratios and broad enrichment patterns. Using publicly available datasets we showed that WaveSeq compares favorably with other published methods, exhibiting high sensitivity and precision for both punctate and diffuse enrichment regions even in the absence of a control data set. The application of our algorithm to a complex histone modification data set helped make novel functional discoveries which further underlined its utility in such an experimental setup.

**Conclusions:**

WaveSeq is a highly sensitive method capable of accurate identification of enriched regions in a broad range of data sets. WaveSeq can detect both narrow and broad peaks with a high degree of accuracy even in low signal-to-noise ratio data sets. WaveSeq is also suited for application in complex experimental scenarios, helping make biologically relevant functional discoveries.

## Background

Chromatin immunoprecipitation followed by massively parallel sequencing (ChIP-Seq) is a powerful experimental framework that enables genome-wide detection of epigenetic phenomena such as histone modifications. Histone modification profiles have diverse characteristics ranging from sharp well-defined peaks surrounding transcription start sites of genes to broad diffuse marks on large genomic regions. This inherent variability makes it difficult to distinguish regions of true enrichment from background noise.

There have been several attempts at solving the problem of finding statistically enriched peaks in ChIP-Seq data. One class of methods focuses on transcription factor ChIP-Seq experiments and uses various features of the data to predict binding regions. For instance, FindPeaks [1] adopts a height threshold together with a simulated random background to find significant peaks, while MACS [2] uses a local Poisson p-value to detect chromatin enrichments. Most of these methods have comparable sensitivity in detecting transcription factor binding sites (TFBSs) and are often used in conjunction with motif-finding algorithms.

While the success of the above set of methods in finding transcription factor binding patterns from ChIP-Seq data is undeniable, histone modification data pose new challenges. Utilization of local features to detect histone modification peaks is difficult due to the relative diffuseness of enrichment patterns. Also, common assumptions of such analyses may not hold in this case. For instance, TFBSs cover a small proportion of the genome, but certain histone marks can be present on much larger genomic fractions. A combination of such factors has led to a relative paucity of methods to analyze histone modification data. A commonly used tool, SICER [3], fits a Poisson distribution before employing kernel density estimation to cluster enriched regions, while a recent study employed a negative binomial regression framework and incorporated genomic covariates to improve ChIP-Seq peak detection [4]. However, with the discovery of an ever-increasing number of histone marks that encompass a wide variety of enrichment patterns, there is a continuing need for improved methods robust to a range of data characteristics.

Wavelets belong to a class of spectral analysis techniques that can extract meaningful information from data by decomposing it into its underlying patterns. The versatility of wavelets has seen them being used in a wide variety of disciplines ranging from image processing to medical diagnostics. Recently, we applied this technique to the analysis of comparative genomics hybridization data [5], utilizing the wavelet property of global pattern quantification to find evolutionary relationships between copy-number profiles in human and bovine populations. However, wavelets also have excellent spatial resolution and comparing data sets one can not only find differences in frequencies of global patterns but also the precise locations of such variations. This property is highly desirable for genome-wide analyses and is the primary motivation for this work.

We present WaveSeq, a novel data-driven method of ChIP-Seq analysis that utilizes the wavelet power spectrum to detect statistically significant peaks in ChIP-Seq data having punctate or broad enrichment patterns. WaveSeq employs Monte Carlo sampling in the wavelet space to predict regions of true enrichment in ChIP-Seq data. In the absence of a control, a randomized algorithm constrained by the length distribution of putative peaks is used to estimate the background read distribution and predict regions of significant enrichment. The non-parametric modeling approach ensures that WaveSeq is robust to variations in data characteristics (e.g. genome coverage) and produces accurate peak calls for a wide variety of data types.

WaveSeq was applied to ChIP-Seq data of Growth-associated binding protein (GABP), Neuron restrictive silencing factor (NRSF) and trimethylations of histone H3 at lysine 4 (H3K4me3), lysine 27 (H3K27me3) and lysine 36 (H3K36me3), which were chosen to encompass a wide variety of enrichment patterns and signal-to-noise ratios (SNRs). We demonstrated that WaveSeq peak calls have high sensitivity and precision for narrow and broad regions over a range of SNRs even in the absence of a control data set. We further exhibited the utility of our approach in a complex experimental setting by analyzing H3K4me3 data from genetically similar chicken lines that exhibit divergent responses to a cancer-causing virus. Differentially marked regions detected by WaveSeq revealed functional differences between the lines that could contribute to differences in disease prognosis. Thus, we conclude that WaveSeq is a highly sensitive algorithm for ChIP-Seq analysis, with applicability for a diverse range of enrichment patterns.

## Results

### Wavelets for ChIP-Seq Analysis

**Figure 1:**
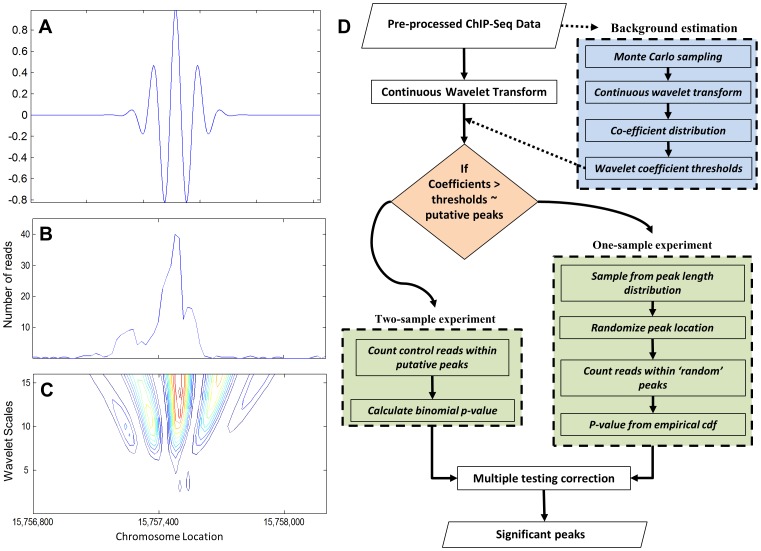
WaveSeq utilizes the continuous wavelet power spectrum to detect peaks in ChIP-Seq data. (a) A scaled representation of the morlet wavelet. (b & c) H3K4me3 data and a contour plot of the associated wavelet power spectrum shows hot spots that correlate with ChIP enrichments. The ChIP-Seq data represents the 15,756,800–15,758,200 bp region of the mouse chromosome 1 from the MEF H3K4me3 data set. (d) A schematic of the WaveSeq analysis pipeline. The workflow consists of two major modules: (i) the Monte Carlo background estimation step and (ii) significance estimation from randomized algorithm using the peak length distribution (one-sample experiment) or an exact binomial test (two-sample experiment).

The wavelet transform has great utility in data compression and pattern finding, the latter involving the choice of a suitable ‘mother’ wavelet *ψ* to best capture underlying patterns in the data. An example of a mother wavelet is the Morlet wavelet, defined as the product of a Gaussian envelope and a cosine wave:
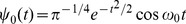
where, *t* is the genomic location and 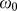 is the non-dimensional frequency (Figure 1A). The wavelet transform may be either continuous or discrete – the continuous wavelet transform (CWT) is highly redundant and resistant to data loss while the discrete transform is less computationally intensive but more prone to information loss. The peaks observed in ChIP-Seq data are relatively smooth, making it better suited to the application of the CWT.

The CWT consists of the convolution of a translated and scaled mother wavelet 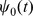 to the signal 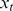 at a predefined step-size (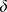) as follows:
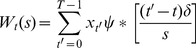
where, (*) indicates the complex conjugate, *s* is the wavelet scale and *t’* denotes translation along the genome. The wavelet scale *s* is representative of the size of the scaled wavelet and the mathematical formulation of the transform implies an inverse relationship, i.e. the higher the scale, the smaller the scaled wavelet. The wavelet decomposition produces a series of ‘wavelet coefficients’, real numbers that indicate the correlation between the mother wavelet and the data, which may be either positive or negative. This is also a multi-scale decomposition, i.e. the coefficients at different scales represent the correlation of scaled versions of the wavelet to the signal. Therefore, smaller localized patterns are likely to be captured by higher scales of the transform and vice-versa.

A natural way of quantifying the wavelet decomposition is the wavelet power spectrum, defined as the square of the wavelet coefficients, and synonymous with the ‘energy density’. A contour plot of the wavelet power spectrum for ChIP-Seq data revealed hot-spots that correlated with peaks (Figures 1B, C). This suggested that wavelets could be used to detect enrichment regions in this type of data and inspired us to use this approach for ChIP-Seq analysis.

### WaveSeq Overview

We introduce WaveSeq, a novel method of ChIP-Seq peak detection that utilizes the wavelet power spectrum (Figure 1D). Sequence reads are first ‘shifted’ to represent the center of DNA fragments obtained from the ChIP experiment. The genome is divided into non-overlapping windows and read counts for each window calculated. The summary read counts are the primary input data format used by WaveSeq. Typical analyses can be of two types: (i) single sample experiment – without control, and (ii) two-sample experiment – with matched control samples.

For both analyses, we first employ a Monte Carlo sampling technique for modeling the data [6]. *N* random samples are drawn from the ChIP-Seq data and the wavelet power calculated for each instance. A slice of the power spectrum at a fixed point of each random sample is used to generate an empirical distribution of wavelet powers for each scale. This distribution enables us to obtain a suitable significance threshold which is applied to the wavelet transform of read count profiles to detect windows having significant enrichment. Our thresholding procedure is, therefore, dependent on the *wavelet fit* to the data at a particular position and distinct from a simple read-count cutoff.

To further account for broad peaks seen in histone modification data, our algorithm implements a ‘gap’ parameter, *g*. We define a ‘gap’ as a window having a non-significant wavelet power (non-significant window); for example, if *g* is set to two, peaks separated by at most two non-significant windows are aggregated together. This parameter is necessary for two reasons: (i) chromatin enrichments, especially broad marks, such as, H3K36me3 and H3K27me3, can be discontinuous and (ii) wavelets are very sensitive to boundary events and local fluctuations. A strong enrichment region interspersed with areas of low read counts could, therefore, result in multiple peak calls and the gap parameter of WaveSeq helps to reduce the effect of this scenario. This parameter is similar in principle to that used in SICER, but with one major distinction. SICER also imposes an upper limit on allowable non-significant windows within a significant peak. While this results in an elegant closed form expression for estimating statistical significance from the score distribution, in practice, this results in smaller peak lengths for the same value of *g* (Figure S1).

#### One-sample experiment

The estimation of statistical significance is crucial to ChIP-Seq analysis approaches to filter the results of genome-wide studies, particularly in the absence of a control. For a single-sample experiment, WaveSeq utilizes the length distribution of putative peaks to estimate the likelihood of observing a peak with a given number of reads.

A large number of peaks, *P*, are sampled with replacement from the length distribution of putative peaks, and their positions on the genome randomized. The number of reads within each randomized peak is counted, generating the empirical distribution, *F(R)*, for the number of peaks having a given read count *R*. The probability of observing a peak with read count *r* is:
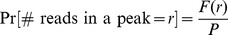
and the p-value of observing this peak is,



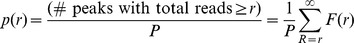



The p-values are subsequently corrected for multiple-testing using the Benjamini-Hochberg FDR procedure [7].

Most ChIP-Seq experiments produce sparse enrichment regions covering a small fraction of the genome and therefore, only few of the randomized peak locations would be likely to overlap significantly enriched regions. However, this is not always the case – histone modifications such as, H3K27me3, mark large regions for silencing and could occupy a significantly greater genomic fraction. In the latter case, a higher proportion of randomized peaks would potentially overlap ‘true’ enrichment regions – but this is a fair reflection of a relatively low SNR data set where the boundaries between true signal and background are blurred.

Thus, it is important to note that in predicting areas of true enrichment in ChIP-Seq data, we do not make any assumptions about the read distribution, instead relying on a sampling technique constrained by the peak length distribution to predict enriched regions. In addition, the association of statistical significance of a peak with its read count provides a natural and interpretable criterion for thresholding genome-wide analyses where the number of reads mapping to a region is often indicative of the presence of a true biological signal.

#### Two-sample experiment

If a ChIP-Seq experiment has matched controls, WaveSeq uses the binomial distribution to compare read counts between normalized test and control samples. For each putative peak, reads in the corresponding region of the control data (*C*) are counted and compared to the test sample (*T*) using a two-sided exact binomial test. A putative peak can be considered to be a Bernoulli experiment with *t* = (*C* + *T*) trials wherein the number of reads in the test sample *T* is the number of successes. The proportion of successes, *p = T*/(*C+T*) and failures, *q* = 1– *p*. In this case, the probability of observing at least *T* successes in *t* trials under the null hypothesis, H_0_: *p* = 0.5, is given by the expression,
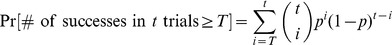



The p-values for the list of putative peaks are subsequently corrected for multiple testing as above [7].

#### Choice of parameters

The key parameters controlling WaveSeq performance are the choice of mother wavelet, size of the sample for background estimation, p-value for determining wavelet power thresholds (*p_thres_*) and gap size *g*. We have tested several wavelets on ChIP-Seq data and found that the morlet wavelet is well suited for detecting punctate and broad peaks less than 10 kb as observed in TFBS, H3K4me3 and H3K36me3 data while the Mexican hat wavelet appears more suitable for calling very broad marks (>10 kb) seen in H3K27me3. The choice of sample size for the Monte Carlo background estimation procedure can impact sensitivity and running time. However, the effects of the above factors appear to be minimized at a sample size of 2^12^.

The user has further control over the peak calling behaviour of WaveSeq with the parameters *p_thres_* and *g*, larger values of both resulting in greater peaks lengths called by WaveSeq. There is a subtle effect of adjusting *p_thres_* as this effectively lowers the stringency in determining the wavelet power thresholds for the peak-calling step. A strong, localized signal can be identified with a lower value of this parameter (e.g. *p_thres_* = 0.2), while the detection of broader peaks may require a looser criterion (e.g. *p_thres_* = 0.4). The choice of a suitable gap size is dependent upon multiple factors including histone mark characteristics and sequencing depth. The read coverage fractions for different histone marks appear to saturate with increasing gap sizes (Figure S2). However, the saturation rate is highly variable between marks - H3K4me3 shows little change with increasing gap sizes, H3K27me3 exhibits a gradual increase while the pattern for H3K36me3 is intermediate between the two, in keeping with the intermediate characteristics of the mark. The above comparison shows that a gap size of 0 to 400 bp (0–2 200 bp windows) would be suitable for the H3K4me3 data set while larger gap sizes may be more appropriate for the broader histone marks e.g. *g* = 5 for H3K36me3 and *g* = 10 for H3K27me3. A similar comparison of read coverage saturation rates can, therefore, help the user choose a gap size appropriate for a particular data set.

### Comparison with other Methods using Published Data

Recent studies have compared the performance of several published ChIP-Seq peak calling algorithms [8], [9]. From the list of methods tested in the above studies, we chose five commonly used tools: FindPeaks, MACS and SiSSRs [10], which were developed primarily for detecting transcription factor binding sites (TF-methods) along with SICER and RSEG [11] which were specifically aimed at chromatin enrichment data (CH-methods). A variety of ChIP-Seq data sets were selected to compare the performance of WaveSeq with the above methods including GABP, NRSF [12], H3K4me3, H3K27me3, H3K36me3 [13] and a synthetic spike-in data set [14].

#### WaveSeq has high sensitivity

**Figure 2:**
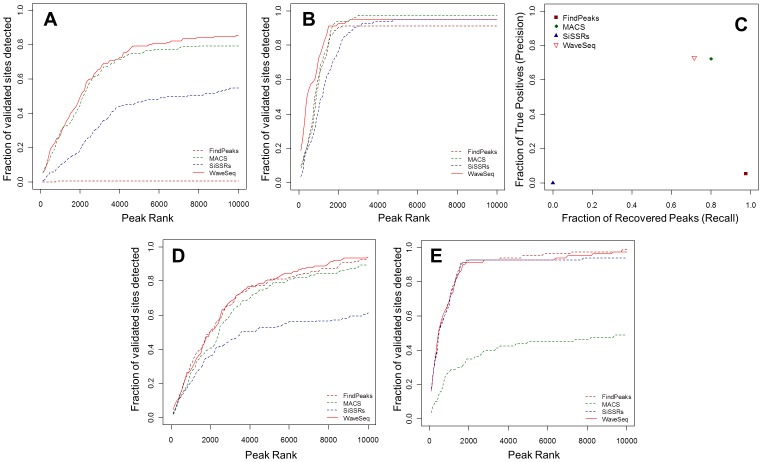
WaveSeq has high sensitivity and precision for punctate data sets. (a & b) Plots of peak ranks against the fraction of validated sites detected by WaveSeq, FindPeaks, MACS and SiSSRs for the (a) GABP and (b) NRSF data sets. WaveSeq has the highest sensitivity for the GABP data set closely followed by MACS, while all methods performed comparably for the NRSF data. (c) A plot of the fraction of true positives (Precision) against the fraction of recovered peaks (recall) for the synthetic spike-in data set shows MACS has the best combination of the two, closely followed by WaveSeq. FindPeaks calls a large number of false positives while SiSSRs fails to detect any peaks. (d & e) Sensitivity plots for the (d) GABP and (e) NRSF data sets shows that WaveSeq has high sensitivity for these data sets even in the absence of control. FindPeaks performs much better on these data sets without control and has almost identical sensitivity as WaveSeq. SiSSRs has mixed results with low sensitivity for GABP and high for NRSF while the reverse is true of MACS.

Several GABP and NRSF binding sites have been validated with qPCR [9] allowing us to compare the sensitivities of the TF-methods with that of WaveSeq using the corresponding ChIP-Seq data. The peaks called by each TF-method were ranked by significance scores output by the method and tested for overlap with the validated sites. Subsequently, we plotted the peak rank against the fraction of validated sites detected by each algorithm (Figures 2A, B).

WaveSeq had the highest sensitivity among tested methods for both data sets. In the case of GABP, WaveSeq had the best performance closely followed by MACS which had slightly lower recall. SiSSRs came in third but still significantly outperformed FindPeaks which had low sensitivity for this data set. On the other hand, all the methods had similar performance on the NRSF data. WaveSeq showed marginally higher sensitivity with MACS, FindPeaks and SiSSRs performing comparably. A further comparison of peak lengths showed that MACS, FindPeaks and WaveSeq had similar peak length distributions while a majority of SiSSRs peaks were very small (Figure S3).

#### WaveSeq has good precision

It is difficult to evaluate the specificity of ChIP-Seq peak-calling algorithms due to the unavailability of adequate ‘true-negative’ binding sites for systematic analysis. However, one can estimate the false positive rates using synthetic data sets which contain simulated binding events. For this analysis we utilized a published synthetic data set generated from human input control data that was ‘spiked’ with simulated reads at fixed locations [14]. We applied WaveSeq and the TF-methods to this data set and plotted the proportion of recovered peaks (recall) against the fraction of true positives (precision) (Figure 2C).

MACS had the best combination of precision (0.724) and recall (0.799), closely followed by WaveSeq which had slightly better precision (0.728) but lower recall (0.716). However, FindPeaks had a very high number of false positives (precision = 0.06) in this test while SiSSRs failed to detect any peaks.

#### WaveSeq performs well even without a control data set

The data from a matched input control sample is considered to improve the power of a ChIP-Seq experiment by reducing systematic biases [15]. However, matching input controls are often not available and negative controls such as IgG that bind in a non-specific manner, can give rise to additional sources of error. Moreover, it is not clear if the use of input alone can offset the effect of various confounding factors such as mappability and G/C content. Therefore, it is important to assess the performance of ChIP-Seq peak callers in the absence of a matched control.

We compared the sensitivity of TF-methods and WaveSeq using the GABP and NRSF data sets as above, but without the use of control data (Figures 2D, E). WaveSeq again had high sensitivity for both data sets, almost identical to FindPeaks which performed much better on these data sets without control. SiSSRs and MACS had mixed results; the former had similar performance to FindPeaks and WaveSeq for the NRSF data set, but lower sensitivity for the GABP data, while the situation was reversed for MACS. Thus, WaveSeq was shown to have high accuracy for punctate peaks and was the only method that performed consistently well for the tested data sets.

#### WaveSeq improves detection of broad histone modification peaks

A lack of adequate validated sites for histone modification data makes it difficult to assess the performance of analysis methods on these data sets. However, we can argue that if multiple methods of analysis based on different detection algorithms predicted significant enrichment in a particular region, it was more likely that a true region of enrichment existed in that region. Indeed, studies have shown that a smaller number of peaks generated by certain methods were largely contained within larger peak lists called by other methods, indicating a common set of peaks detected by most algorithms [9]. With the above intuition we ran the CH-methods on the MEF histone modification data sets. We included MACS in the latter as it has been used for broad peak calling [16], even though it was originally developed for the analysis of transcription factor ChIP-Seq data. The top peaks (15000 for H3K4me3 and 20000 for H3K36me3 and H3K27me3) called by each of the above programs were compared and regions detected by at least two peak-callers were defined as putative ‘true positives’. When calculating putative true positive peaks, we did not enforce any restrictions on the overlap, i.e. if there was even a single bp overlap between two peak calls, these regions were merged together (union) into a putative positive peak. This is because peak-calling algorithms will sometimes call only a part of a putative histone modification enrichment as a peak, and merging adjacent peak-calls is likely to produce a better reflection of enrichment patterns.

**Figure 3:**
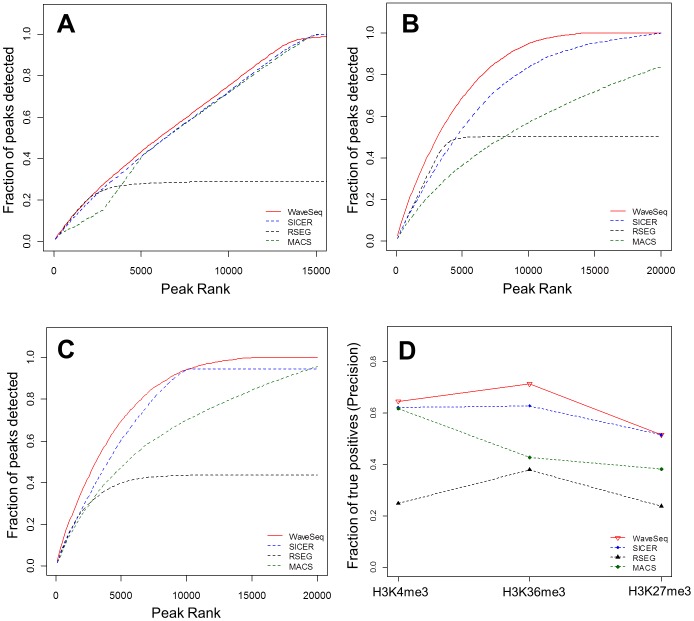
WaveSeq improves detection of histone modification peaks. (a, b & c) Plots of peak ranks against the fraction of putative ‘true positive’ sites detected by WaveSeq, SICER, RSEG and MACS for the (a) H3K4me3, (b) H3K36me3 and (c) H3K27me3 data sets. All methods apart from RSEG perform comparably on the punctate H3K4me3 data. However, WaveSeq outperforms the other methods on the broader peaks of H3K36me3 and H3K27me3. SICER comes in second while MACS has low sensitivity for diffuse data. RSEG has good sensitivity for the strongest peaks but suffers from low recall, failing to detect any peaks in chromosomes 10–19. (d) A plot of the fraction of true positives (precision) from the top 10000 peaks detected by the above four methods in the MEF histone modification data sets shows that WaveSeq has the best performance, closely followed by SICER. MACS performs well only on the H3K4me3 data while RSEG has low precision for all the three data sets.

The above procedure yielded 8592, 7522 and 5463 peaks for the H3K4me3, H3K36me3 and H3K27me3 data sets, respectively. These peaks were compared with the peak lists from all methods (SICER, RSEG, MACS and WaveSeq) and relative performance was assessed by comparing the fraction of recovered peaks against peak ranks (Figures 3A–C). For punctate H3K4me3 data, all methods apart from RSEG performed well, with near-identical recall rates. WaveSeq had the best sensitivity on the H3K36me3 and H3K27me3 data sets with SICER coming in second. MACS showed lower recall rates for these two data sets while RSEG detected the top peaks with good accuracy but was unable to detect any peaks in chromosomes 10–19. A further analysis of precision (Figure 3D) showed that WaveSeq had the highest performance in all three data sets.

Pair-wise comparisons between peaks detected by WaveSeq and those called by SICER and MACS showed a high degree of overlap (98–100%) across all the data sets. In the case of RSEG the overlap was lower (20–68%) but closer examination revealed that a majority of regions not called by WaveSeq, particularly in the H3K4me3 and H3K36me3 data sets, had low average read counts and were possibly false positives (Figure S4). WaveSeq also called larger peaks on average compared to SICER, particularly in the H3K27me3 and H3K4me3 data sets (Figure S1). However, RSEG detected very broad regions in both H3K27me3 and H3K4me3 data. Since this algorithm was developed with the express purpose of detecting dispersed chromatin domains, the above behaviour is expected, although very long peaks in punctate ChIP-Seq data may not be desirable. Also, somewhat surprisingly, WaveSeq and SICER had greater average peak lengths compared to RSEG for the H3K36me3 data. MACS, on the other hand, detected very small peaks in all the data sets, proving its general unsuitability for broad histone marks.

Thus, WaveSeq once again showed the highest sensitivity of all tested methods across a variety of histone modification data sets. While there was little to choose between the different algorithms for the punctate high SNR H3K4me3 data, WaveSeq outperformed the other tested methods in the analysis of broad enrichment regions characteristic of broad marks such as H3K27me3 and H3K36me3.

### Analysis of Complex Histone Modification Data

The bursa of Fabricius is a specialized immune organ that is the site of haematopoiesis and B cell development in chickens. This tissue is one of the first targets of Marek’s disease virus (MDV), a herpesvirus that induces T-cell lymphomas in susceptible birds. Genetically similar lines of chickens that show differential resistance to Marek’s disease (MD) have been developed and studied for decades, but the exact causes of the divergent response have not been found, although it is believed that epigenetic factors play an important role in determining the level of resistance of an individual. This is an interesting epigenetic model for human cancers as individuals having high genetic similarity exhibit natural resistance to a cancer-causing agent. Moreover, this is a complex ChIP-Seq experiment representing studies in non-traditional systems that are becoming more prevalent with the plummeting costs of sequencing. To demonstrate the utility of WaveSeq in such an experimental scenario we used it to analyze H3K4me3 profiles in matched infected and control birds from inbred chicken lines having diverse responses to MD.

#### WaveSeq detects differential H3K4me3 marks induced by virus infection

We generated H3K4me3 ChIP-Seq data from inbred chicken lines – line 6_3_ is highly resistant while line 7_2_ is highly susceptible to MD – in matched infected and control groups. In the subsequent discussion, we refer to the resistant line 6_3_ and susceptible line 7_2_ as R and S groups, respectively. We first analyzed the infected group with the non-infected group as control. The samples were then swapped to account for significant peaks in the control that were absent in the infected group. This is in contrast to traditional ChIP-Seq experiments where peaks detected in an input control represent false positives and are removed from subsequent analyses. Statistical significance for differentially marked regions (DMRs) was defined at a false discovery rate of 5% (FDR <0.05). DMRs were compared across the control-swapped comparisons and merged into a single non-redundant list.

WaveSeq detected a comparable number of peaks in the two groups, with 25050 and 27169 peaks in the R and S groups, respectively. The resistant line did not show any differential H3K4me3 marks at the predefined significance level. In contrast, there were 310 H3K4me3 DMRs in the susceptible line, all but five of which were more enriched in infected individuals. This confirmed the presence of dramatic differences in the epigenetic effects of MDV on the two lines, with a predominantly activating effect of the virus infection.

#### Increased B cell activation in susceptible birds as a result of MD

To investigate the functional implications of observed epigenetic differences, we searched for overlaps between H3K4me3 DMRs and gene promoters and were able to map 241 regions to 310 Ensembl genes (Table S1). Functional annotation of these genes with DAVID [17], [18] revealed significant enrichment of various immune-related functions, such as, hemopoeisis, positive regulation of lymphocyte activation, response to DNA damage stimulus and regulation of apoptosis (Table 1). Thus, there appeared to be a significant activation of the immune system in infected birds of the S group, consistent with the observed response at the early cytolytic stage of the disease in susceptible birds. Moreover, 81 of these genes (26.6%; p = 5.8×10^−7^) had reported expression in bursa (Table S2).

**Table 1:** Functional annotation of genes having H3K4me3 DMRs.

Gene Ontology Term	Count	p-value	FDR (%)
GO:0002520: Immune system development	15	1.91×10^−8^	3.02×10^−5^
GO:0030097: Hemopoiesis	14	2.16×10^−8^	3.41×10^−5^
GO:0048534: Hemopoietic or lymphoid organ development	14	8.76×10^−8^	1.38×10^−4^
GO:0045580: Regulation of T cell differentiation	7	8.60×10^−7^	0.001359
GO:0002521: Leukocyte differentiation	10	1.11×10^−6^	0.001747
GO:0045582: Positive regulation of T cell differentiation	6	1.23×10^−6^	0.001951
GO:0045321: Leukocyte activation	11	1.70×10^−6^	0.002693
GO:0045619: Regulation of lymphocyte differentiation	7	2.39×10^−6^	0.003781
GO:0002684: Positive regulation of immune system process	10	2.73×10^−6^	0.004309
GO:0045621: Positive regulation of lymphocyte differentiation	6	3.33×10^−6^	0.005262
GO:0046649: Lymphocyte activation	10	4.70×10^−6^	0.007428
GO:0050870: Positive regulation of T cell activation	8	5.53×10^−6^	0.008734
GO:0001775: Cell activation	11	6.17×10^−6^	0.009752
GO:0051251: Positive regulation of lymphocyte activation	8	8.08×10^−6^	0.012774
GO:0002696: Positive regulation of leukocyte activation	8	1.16×10^−5^	0.018257
GO:0050867: Positive regulation of cell activation	8	1.62×10^−5^	0.025558
GO:0050863: Regulation of T cell activation	8	1.62×10^−5^	0.025558
GO:0030098: Lymphocyte differentiation	8	1.90×10^−5^	0.030027
GO:0051249: Regulation of lymphocyte activation	8	2.59×10^−5^	0.040908
GO:0030217: T cell differentiation	7	2.76×10^−5^	0.04356
GO:0002694: Regulation of leukocyte activation	8	4.00×10^−5^	0.063158
GO:0045058: T cell selection	5	6.65×10^−5^	0.105094
GO:0050865: Regulation of cell activation	8	6.80×10^−5^	0.107401
GO:0002252: Immune effector process	6	1.38×10^−4^	0.218176
GO:0033077: T cell differentiation in the thymus	5	2.30×10^−4^	0.362727
GO:0042110: T cell activation	7	2.43×10^−4^	0.38295
GO:0042981: Regulation of apoptosis	14	2.47×10^−4^	0.389793
GO:0043067: Regulation of programmed cell death	14	2.98×10^−4^	0.469488
GO:0010941: Regulation of cell death	14	3.12×10^−4^	0.491456
GO:0033554: Cellular response to stress	12	4.00×10^−4^	0.629557
GO:0045061: Thymic T cell selection	4	4.06×10^−4^	0.639966

The top functional categories (FDR <1%) enriched among genes having H3K4me3 DMRs from DAVID shows a large number of immune-related functions. Count refers to the number of genes in the gene list annotated with the given GO ID. P-values were obtained from a modified Fisher exact test performed by DAVID which tests the enrichment of the corresponding functional category in the given gene list against the population (chicken genome). FDR correction was performed using the Benjamini-Hochberg procedure [7].

**Figure 4:**
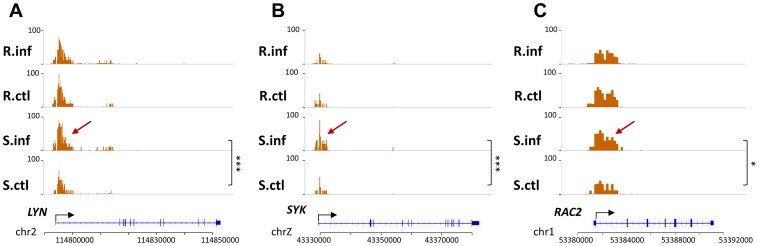
Differentially marked regions detected by WaveSeq suggest increased B cell activation in susceptible chickens. Several genes involved in the B cell activation such as *LYN* (a), *SYK* (b) and *RAC2* (c) show increased levels of H3K4me3 in infected birds from the S group as shown by the arrowheads. In contrast, there are no significant changes in the R group. This suggests the presence of increased numbers of activated B cells in susceptible birds that may lead to increased viral loads in latter stages of MD. *** = p<0.001; * = p<0.05. S.inf = infected S group, S.ctl = control S group, R.inf = infected R group, R.ctl = control R group.

Several genes having H3K4me3 DMRs were involved in the PANTHER [19] B-cell signalling pathway (p = 1.3×10^−3^) such as *LYN, SYK, GRB2, PTPRC, RAC2* and *BLNK*, indicative of increased B cell activation in the infected S group. The signalling molecules CD45, Lyn and Syk, gene products of *PTPRC, LYN* and *SYK*, respectively, are major players in the early stages of B cell antigen receptor signalling. These genes work together with *BLNK* and *GRB2* to activate B cells via the NF-κB mediated pathway while *BLNK* and *RAC2* may also activate B cells via the ERK, p38 or jun signalling cascades. H3K4me3 levels on all these genes were unchanged in the R group but were significantly higher in the infected S group after MDV infection (Figures 4, S5). Three of these genes – *LYN*, *SYK* and *RAC2*– had reported expression in bursal cells [20] which suggests that the tissue-specific activation of these genes in the bursa might lead to increased B cell activation in susceptible birds.

MDV primarily targets B cells during early stages of the disease as these cells provide the first line of defence via the host humoral immune response. B cells surround the invading virus particles and have increased rates of infection and atrophy. The infection of B cells, in turn, induces the activation of CD4+ T cells which consequently become more vulnerable to virus infection [21]. The increase in B cell activation indicated by elevated levels of H3K4me3 on key genes involved in the pathway suggests the presence of an increased number of activated B cells in susceptible birds and a possible increase in the number of activated CD4+ T lymphocytes. The larger population of cells vulnerable to infection by MDV at the early cytolytic stage of the disease in susceptible birds, could, therefore, result in increased levels of infection and higher mortality in the latter stages of the disease.

## Discussion

The analysis of ChIP-Seq data poses several challenges including a diverse array of enrichment patterns, the lack of true biological controls and confounding factors such as sequencing depth, mappability and G/C content. In the presence of these sources of bias, it is important to have methods of analysis robust to various data characteristics that also preserve prediction accuracy. In response to these issues, we have developed a novel data-driven ChIP-Seq analysis algorithm named WaveSeq which is capable of detecting both punctate and diffuse enrichment regions and is free of distributional assumptions. WaveSeq utilizes non-parametric modeling of ChIP-Seq data using Monte Carlo sampling and a randomized algorithm to accurately estimate the empirical distribution of reads in the absence of a control.

With the aid of a variety of public data sets we were able to demonstrate that WaveSeq has high accuracy and performs favourably in comparison with several published methods of analysis in detecting punctate and diffuse enrichment regions (Figure S6). WaveSeq also performed with comparable accuracy in the absence of control data. Previous studies have observed that the background signal of ChIP-Seq data is non-random [15] and the ability to distinguish regions of true signal from background could be potentially improved if this non-randomness is accounted for. The improved detection capacity exhibited by WaveSeq in the absence of a control data set suggests that the non-parametric modeling approach is successful in capturing the data characteristics leading to higher prediction accuracy.

The rapid advance of epigenetics and the advent of cost-effective next-generation sequencing technologies have led to complex experimental designs being employed to investigate various topics such as the epigenetics of disease response. WaveSeq is capable of being used in such an experimental setting and helps make relevant biological discoveries. We illustrate this by using our algorithm to analyze a complex H3K4me3 data set to investigate the differences in the epigenetic effects of MDV infection in inbred chicken lines having divergent responses to MD. WaveSeq detects the presence of H3K4me3 DMRs on key genes involved in the B cell activation pathway suggesting the presence of increased numbers of activated B cells in infected individuals of the susceptible line. B cells are the primary targets of MDV at the early cytolytic stage of the disease and infection of these cells by the virus leads to activation of CD4+ T cells which are more vulnerable to infection than naive T cells. Consequently, an increase in the number of MDV-infected cells at this stage of the disease could translate to an increased viral load and a worse prognosis in susceptible birds at the latter stages of infection. Thus, epigenetic differences between the two lines could have a major impact on disease progression indicating that epigenetic marks play an important role in regulating disease response.

The absence of distributional assumptions in WaveSeq makes it potentially applicable to other forms of next-generation sequencing data. The detection of the genomic locations of nucleosomes is one such area of current interest. A nucleosome positioning experiment typically consists of the sequencing of DNA fragments associated with mono-nucleosomes across the whole genome. The data consists of broad diffuse regions with peaks that repeat approximately every 147 bp, the length of DNA associated with single nucleosomes. Regions of active transcription have lower nucleosome enrichment while high nucleosome density is associated with silent heterochromatin. Thus, differences in nucleosome density between samples could be predictive of transcriptional differences. Sequencing data having such underlying patterns could be highly suited to the wavelet transform framework employed by WaveSeq.

One of the primary drawbacks of WaveSeq is the relatively high number of peak calls for low SNR data such as H3K27me3 which is an unfortunate side-effect of the sensitivity of the algorithm. However, since peak calls are ranked by FDR, a more stringent criterion can be used to circumvent this issue. Moreover, increased sequencing depth significantly improves discriminative power and is highly recommended particularly for data having diffuse enrichments.

### Conclusions

ChIP-Seq experiments having a wide variety of enrichment patterns and a lack of true biological controls pose significant challenges for analysis and interpretation. WaveSeq is a highly sensitive, data-driven method capable of detecting significantly enriched regions in data having diverse characteristics. WaveSeq can detect both punctate and diffuse regions with a high degree of accuracy even in low SNR data sets. Moreover, it performs with comparable accuracy in the absence of control data. WaveSeq is suited for application in complex experimental scenarios, helping make biologically relevant functional discoveries and compares favourably with existing methods of analysis over a broad variety of data types.

## Materials and Methods

### H3K4me3 Data from Chicken Bursa

Two specific-pathogen-free inbred lines of White Leghorn chickens either resistant (6_3_) or susceptible (7_2_) to MD were hatched, reared and maintained in the Avian Disease and Oncology Laboratory (ADOL, Michigan, USDA). The chickens were injected intra-abdominally with a partially attenuated very virulent plus strain of MDV (648A passage 40) at 5 days after hatch with a viral dosage of 500 plaque-forming units (PFU). Chickens were terminated at 5dpi to collect bursa tissues. All procedures followed the standard animal ethics and use guidelines of ADOL.

ChIP was carried out using bursa from MDV infected and controls birds. About 30 mg bursa samples were collected from three individuals, cut into small pieces (1 mm^3^) and digested with MNase to obtain mononucleosomes. PNK and Klenow enzymes (NBE, Ipswich, MA, USA) were used to repair the ChIP DNA ends pulled down by the antibody. A 3′ adenine was added using Taq polymerase and Illumina adaptors ligated to the repaired ends. Seventeen cycles of PCR was performed on ChIP DNA using the adaptor primers and fragments with a length of about 190 bp (mononucleosome + adaptors) were isolated from agarose gel. Subsequently, cluster generation and sequencing using the purified DNA was performed on the Illumina Genome Analyzer IIx following manufacturer protocols. Sequence reads of length 25 bp were aligned to the May 2006 version of the chicken genome (galGal3) using bowtie version 0.12.7 [22]. Default alignment policies of bowtie were enforced. The antibodies used and the total number of reads obtained for each sample are listed in Table S3.

### Published Datasets used in this Study

We used five ChIP-Seq data sets for benchmarking purposes [12], [13]. The GABP and NRSF (monoclonal) ChIP-Seq data sets were produced from the human Jurkat cell line while a negative control data set was obtained by reverse crosslinking extracted DNA without the subsequent immunoprecipitation step (RX-NoIP). The H3K4me3, H3K27me3 and H3K36me3 data sets were obtained from murine embryonic fibroblast (MEF) cells. We also utilized a previously published synthetic spike-in data set for testing precision and recall [14]. For two-sample ChIP-Seq analyses of GABP and NRSF, we used the RX-NoIP data set as control. The spike-in data consisted of a human input control data set which was randomly divided into three subsets; reads corresponding to the spikes were added to one of the subsets which constituted the test sample while a second subset (without the spike-in reads) served as the control. For the MEF histone modification data no control data sets were used to assess algorithm performance in the absence of control.

### Analysis Parameters

All downloaded data consisted of aligned sequence reads which were converted to the BED format. Redundancies were removed before subsequent analysis. Sequence reads were shifted by 95 bp from the 5′ end to represent the center of the DNA fragments obtained from the nucleosome and the linker DNA (≈ 190 bp). Summary read counts were calculated using non-overlapping windows of 200 bp for visualization and normalized to per million mapped reads in each sample.

Systematic tuning of the WaveSeq peak-calling algorithm was carried out. We used the morlet wavelet for GABP, NRSF, H3K4me3 and H3K36me3 data and the Mexican hat wavelet for the H3K27me3 data. The morlet wavelet showed better energy density within a smaller band of scales and outperformed other tested wavelets in detecting smaller peaks. The Mexican hat wavelet, on the other hand, had a more uniform energy distribution and was better suited to the analysis of broad, diffuse data e.g. H3K27me3. For the Monte Carlo threshold estimation step, we chose *N* = 5000 and a sample length of 2^12^ for optimal accuracy and speed. The sampling was performed chromosome-by-chromosome. The p-value for determining significant enrichments (*p_thres_*) in the GABP, NRSF and H3K4me3 data was chosen to be 0.2 while *p_thres_* = 0.4 worked better with broader peaks (H3K36me3 and H3K27me3). For estimating FDR in one-sample analyses, we used *P* = 10^6^. We set *g* = 0 for the GABP and NRSF data sets while for the MEF H3K4me3 data *g* was chosen to be 2 (400 bp). For the broader peaks of H3K36me3, significant enrichments within 1 kb of each other were aggregated together (*g* = 5), while in the case of H3K27me3 this distance was increased to 2 kb (*g* = 10). All analyses were performed on a 2.66 GHz dual core desktop computer running Windows Vista with 3 GB of RAM, a licensed copy of Matlab v7.4 (R2007a) with the Wavelet Toolbox and R version 2.13.0 [23].

Four methods were chosen for benchmarking: MACS [2] version 1.3.7.1, FindPeaks [1] version 4.0.15, SiSSRs [10] version 1.4, SICER [3] version 1.1 and RSEG [11]. The method parameters used in our analyses are described in Text S1.

### Gene Annotation and Functional Analysis of DMRs

RefSeq and Ensembl gene annotations for the chicken genome (galGal3) were downloaded from the UCSC genome browser [24]. Gene promoters were searched for overlaps with DMRs and all gene names were converted to their Ensembl Ids using the biomart data retrieval system from Ensembl [25], [26]. This unified list of gene Ids was then analyzed for functional annotation enrichment with DAVID [17], [18]. Default parameters were used for DAVID analyses.

### Software Implementation

Data pre-processing, Monte Carlo estimation of wavelet coefficient thresholds and peak-calling modules of WaveSeq were implemented in Matlab. FDR estimation in the presence and absence of control was performed in R. We are currently working on a unified R implementation of the software for public release. WaveSeq can be run on a standard desktop computer with at least 3 GB of RAM and a 2 GHz processor. The software can be used on any species with a sequenced genome. WaveSeq has been tested on Windows and MAC OSX and is currently available for these operating systems from the authors on request.

## Supporting Information

Figure S1 **Peak length distributions of tested methods when applied to histone modification data.** A comparison of peak length distributions for the top 15000 peaks called from the (a) H3K4me3, (b) H3K36me3 and (c) H3K27me3 data. (a) SICER and MACS have similar peak lengths in the H3K4me3 data, followed by WaveSeq. RSEG peak lengths are almost uniformly distributed between 0 and 20 kb. (b) MACS and RSEG called relatively short peaks for H3K36me3 while SICER and WaveSeq detected greater peak lengths. (c) WaveSeq called the longest peaks when applied to H3K27me3 data followed by SICER and RSEG.
(PDF)

Figure S2 **The effect of increasing gap sizes on read coverage of top peaks.** The fraction of reads covered by the top *N* peaks saturates at larger gap sizes. This saturation is almost immediate for H3K4me3, intermediate for H3K36me3 and more gradual for H3K27me3. In the case of H3K4me3, *N = *20000, while for H3K36me3 and H3K27me3, *N = *40000. The window size is 200 bp.
(PDF)

Figure S3 **WaveSeq has comparable peak lengths to MACS and FindPeaks in punctate data sets.** A comparison of peak length densities of the top 20000 peaks for the (a) GABP and (b) NRSF data sets showed comparable peak lengths called by WaveSeq, MACS and FindPeaks. However, SiSSRs consistently calls very small peaks.
(PDF)

Figure S4 **RSEG peaks not detected by WaveSeq have low average read counts and are possibly false positives.** Average read counts within RSEG peaks (a, b & c) and peak length distributions (d, e & f) in the H3K4me3 (a & d), H3K36me3 (b & e) and H3K27me3 (c & f) data. The solid lines correspond to all peaks called by RSEG (All Peaks) and the dashed lines represent those peaks that are not detected by WaveSeq (No overlaps). These plots show that WaveSeq detects a majority of large RSEG peaks in the H3K27me3 and H3K36me3 data. However, most of the H3K4me3 peaks detected by RSEG are very large and appear to be false positives. The average read counts plotted were output by the program.
(PDF)

Figure S5 **Differentially marked regions detected by WaveSeq suggest increased B cell activation.** Several genes involved in the B cell activation such as *PTPRC* (a), *BLNK* (b) and *GRB2* (c) exhibited increased levels of H3K4me3 in infected birds from the S group as shown by the arrowheads. However, there were no significant changes in the R group. ** = p<0.01; * = p<0.05. S.inf = infected S group, S.ctl = control S group, R.inf = infected R group, R.ctl = control R group.
(PDF)

Figure S6 **WaveSeq detects a broad variety of enrichment regions with high accuracy.** Examples of WaveSeq peak calls on MEF histone modification data. (a) WaveSeq detects H3K4me3 and H3K36me3 marks on the housekeeping gene *Polm* located on chromosome 11 and (b) a broad peak of H3K27me3 on the developmental transcription factor *Cdx4* which is silenced in differentiated cell populations.
(PDF)

Table S1 **List of H3K4me3 DMRs and overlapping genes. T**he chromosome, start and end columns refer to the significant DMRs detected by WaveSeq. The columns S.inf and S.ctl contain the normalized reads (per million) mapped to the DMRs in the infected and control samples of the S group, respectively. P-values are calculated by WaveSeq using an exact binomial test and fold change = (S.inf+1)/(S.ctl+1). The columns RefSeq_ID and Ensembl_ID contain RefSeq and Ensembl genes that overlap the corresponding DMRs.
(XLSX)

Table S2 **Genes overlapping H3K4me3 DMRs with reported expression in bursa.** A significant proportion of genes having H3K4me3 DMRs had reported expression in bursa. The annotation was obtained by DAVID from the UniProt database (UP_Tissue). P-values were calculated using a modified Fisher exact test performed by DAVID which tests the enrichment of the corresponding functional category in the given gene list against the population (chicken genome). FDR correction was performed using the Benjamini-Hochberg procedure [7].
(XLSX)

Table S3 **Sequencing results showing the antibody used and raw, mapped and non-redundant read numbers for each sample.** The reads obtained from the chicken bursa H3K4me3 ChIP-Seq experiment. Mapped % and non-redundant % are the ratios of mapped and non-redundant reads to raw reads expressed as a percentage. S.inf = infected S group, S.ctl = control S group, R.inf = infected R group, R.ctl = control R group.
(XLSX)

Text S1 **Supplementary methods.** Parameters used for published algorithms and data access information.
(PDF)
