# Supplementary figures and images for: WaveSeq: A Novel Data-Driven Method of Detecting Histone Modification Enrichments Using Wavelets
Source: PLoS One. 2012 Sep 28;7(9):e45486. doi: 10.1371/journal.pone.0045486 (PMC3461018; doi:10.1371/journal.pone.0045486)

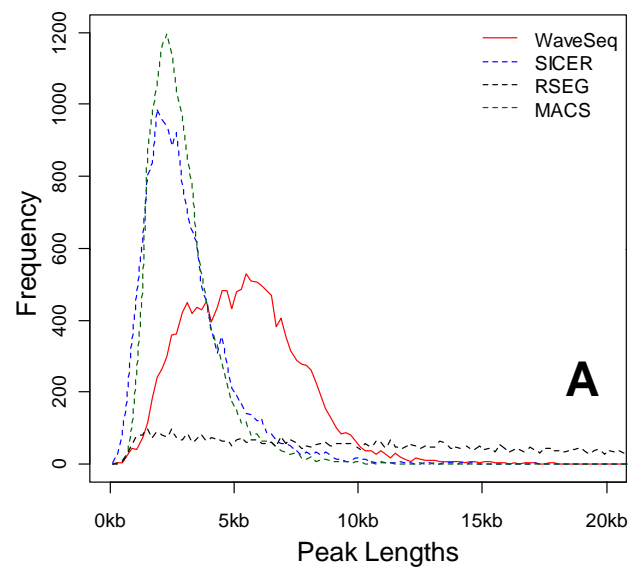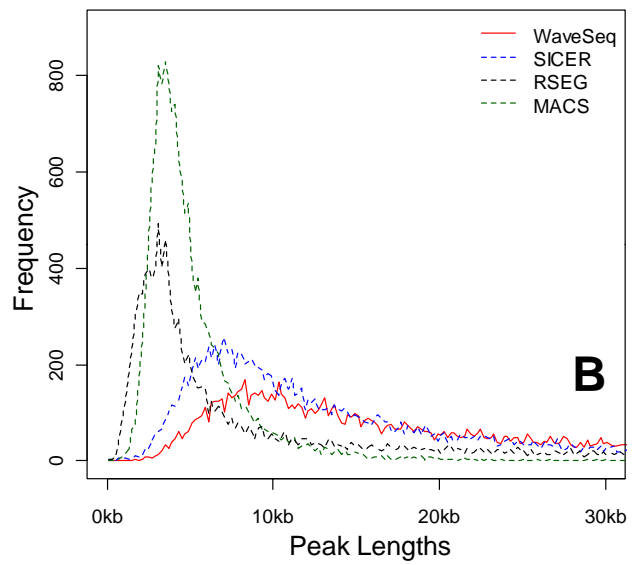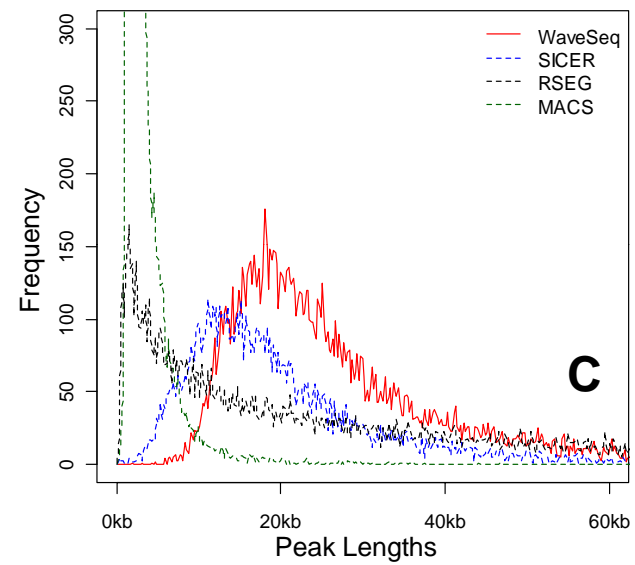

Supplement: Figure S1 — Peak length distributions of tested methods when applied to histone modification data. A comparison of peak length distributions for the top 15000 peaks called from the (a) H3K4me3, (b) H3K36me3 and (c) H3K27me3 data. (a) SICER and MACS have similar peak lengths in the H3K4me3 data, followed by WaveSeq. RSEG peak lengths are almost uniformly distributed between 0 and 20 kb. (b) MACS and RSEG called relatively short peaks for H3K36me3 while SICER and WaveSeq detected greater peak lengths. (c) WaveSeq called the longest peaks when applied to H3K27me3 data followed by SICER and RSEG. (PDF) [file pone.0045486.s001.pdf]

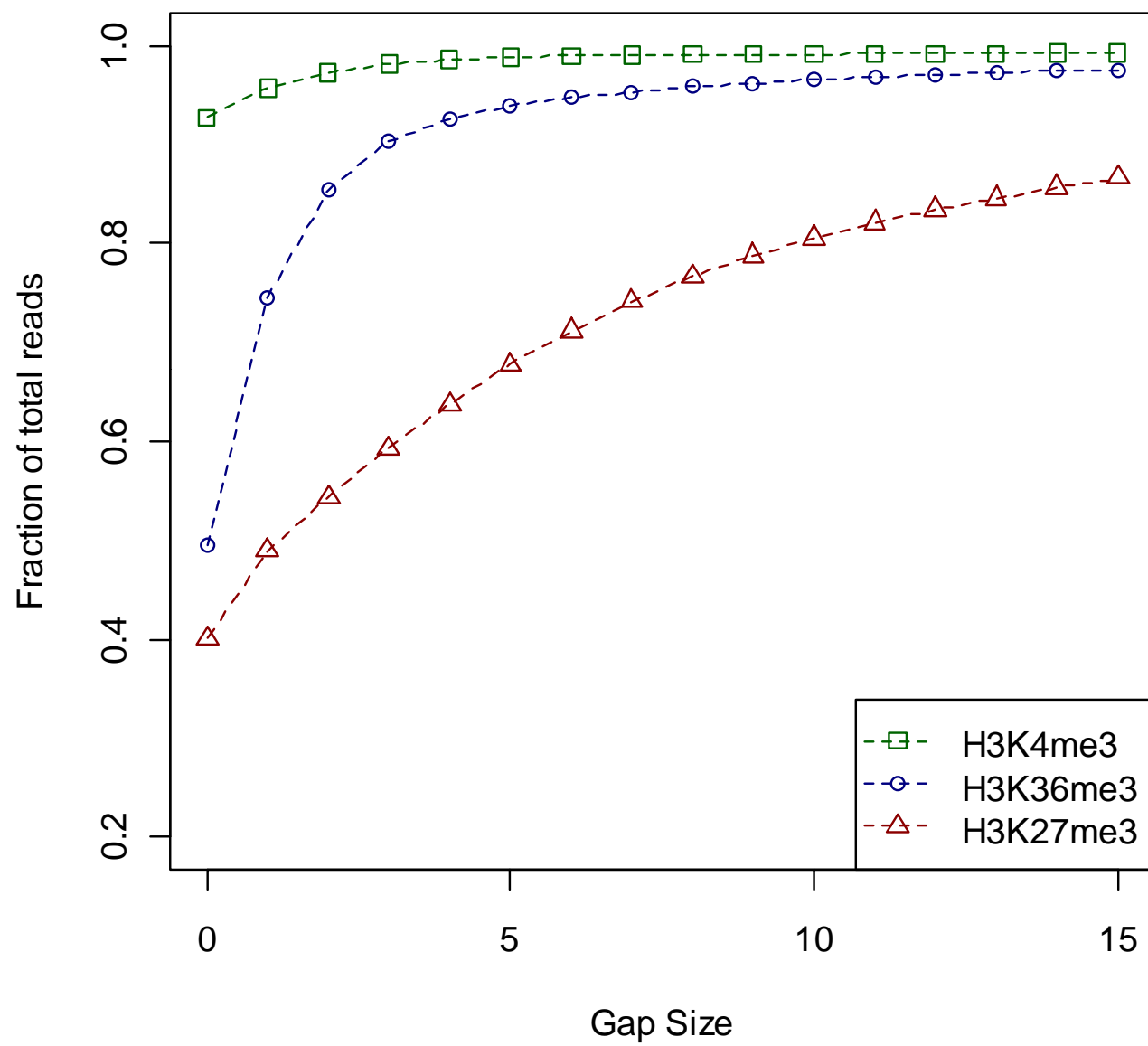

Supplement: Figure S2 — The effect of increasing gap sizes on read coverage of top peaks. The fraction of reads covered by the top N peaks saturates at larger gap sizes. This saturation is almost immediate for H3K4me3, intermediate for H3K36me3 and more gradual for H3K27me3. In the case of H3K4me3, N = 20000, while for H3K36me3 and H3K27me3, N = 40000. The window size is 200 bp. (PDF) [file pone.0045486.s002.pdf]

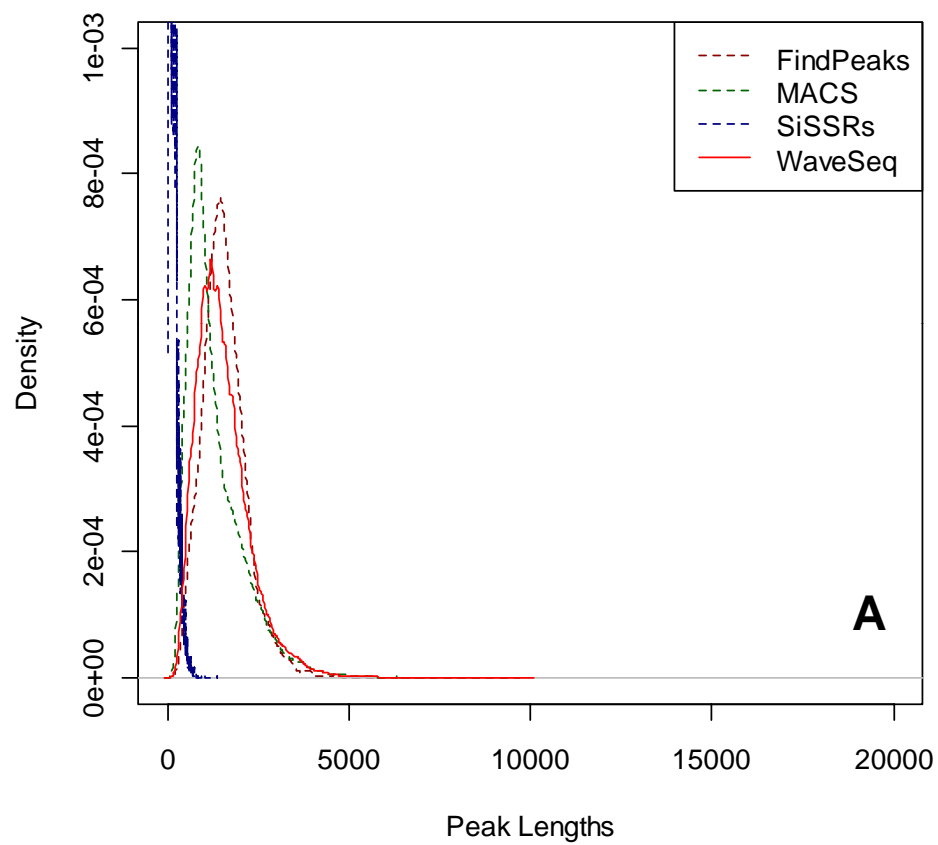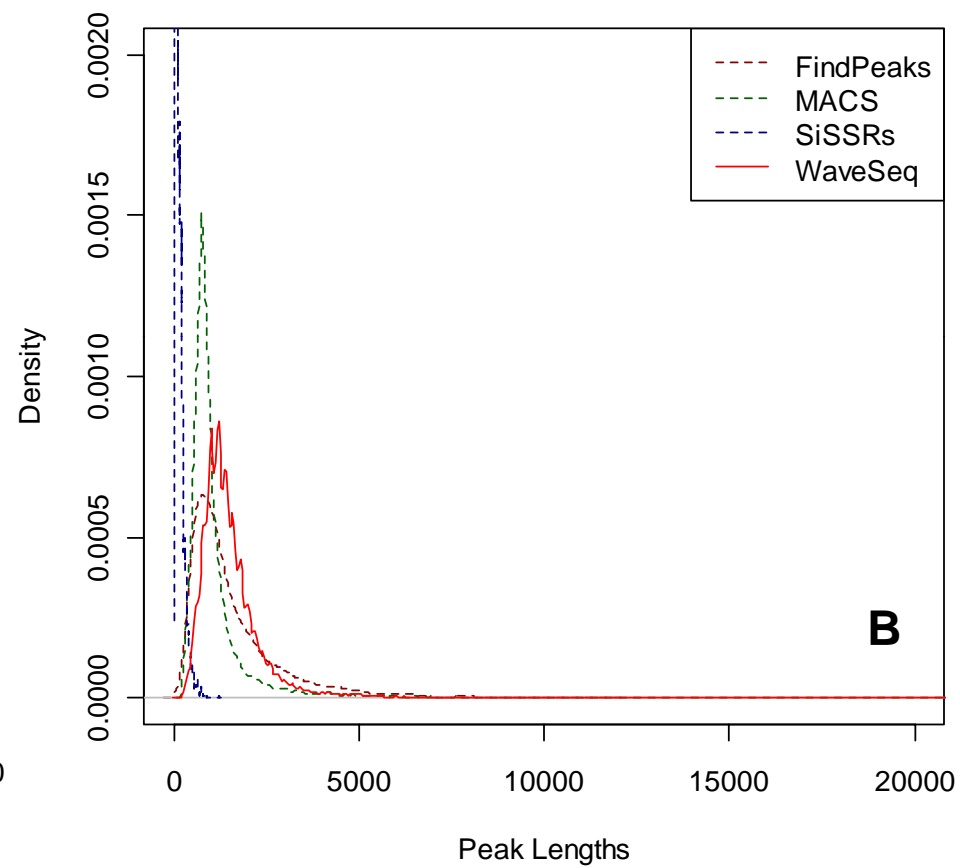

Supplement: Figure S3 — WaveSeq has comparable peak lengths to MACS and FindPeaks in punctate data sets. A comparison of peak length densities of the top 20000 peaks for the (a) GABP and (b) NRSF data sets showed comparable peak lengths called by WaveSeq, MACS and FindPeaks. However, SiSSRs consistently calls very small peaks. (PDF) [file pone.0045486.s003.pdf]

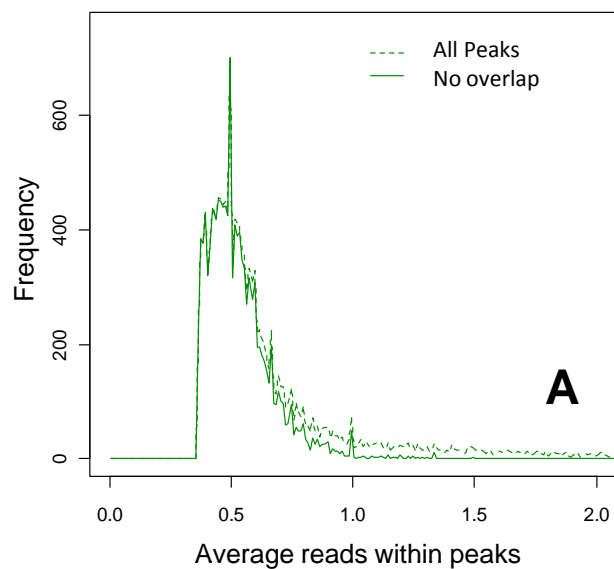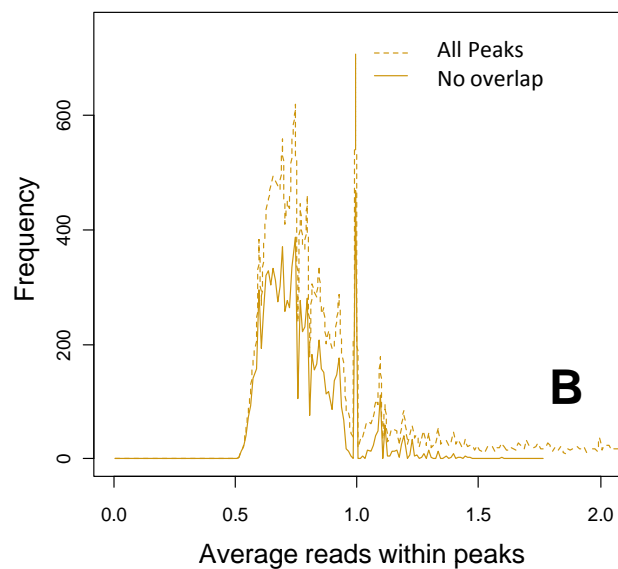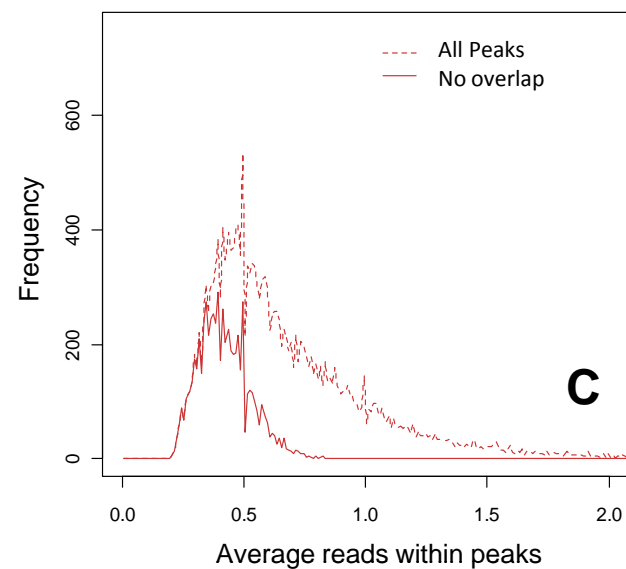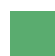

H3K4me3

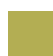

H3K36me3

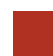

H3K27me3

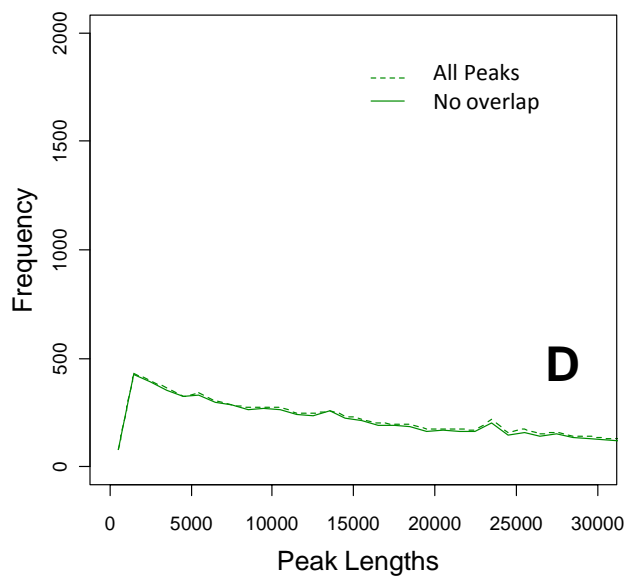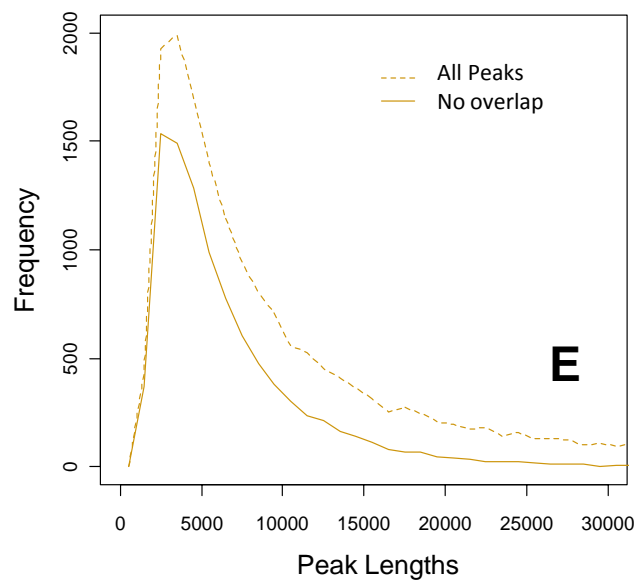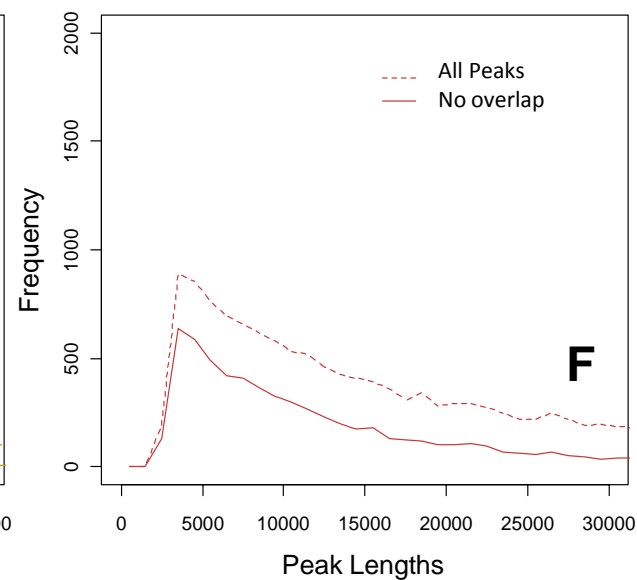

Supplement: Figure S4 — RSEG peaks not detected by WaveSeq have low average read counts and are possibly false positives. Average read counts within RSEG peaks (a, b & c) and peak length distributions (d, e & f) in the H3K4me3 (a & d), H3K36me3 (b & e) and H3K27me3 (c & f) data. The solid lines correspond to all peaks called by RSEG (All Peaks) and the dashed lines represent those peaks that are not detected by WaveSeq (No overlaps). These plots show that WaveSeq detects a majority of large RSEG peaks in the H3K27me3 and H3K36me3 data. However, most of the H3K4me3 peaks detected by RSEG are very large and appear to be false positives. The average read counts plotted were output by the program. (PDF) [file pone.0045486.s004.pdf]

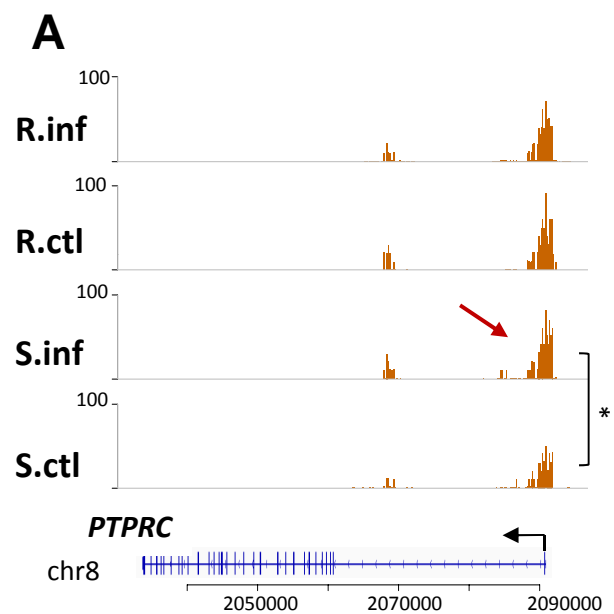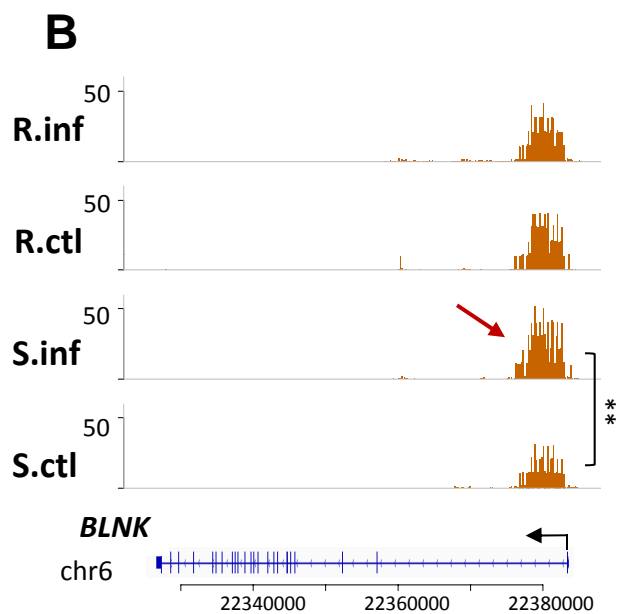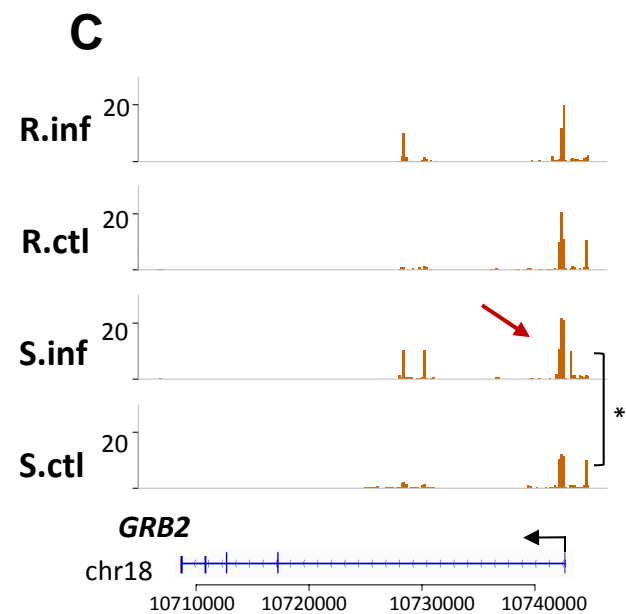

Supplement: Figure S5 — Differentially marked regions detected by WaveSeq suggest increased B cell activation. Several genes involved in the B cell activation such as PTPRC (a), BLNK (b) and GRB2 (c) exhibited increased levels of H3K4me3 in infected birds from the S group as shown by the arrowheads. However, there were no significant changes in the R group. ** = p<0.01; * = p<0.05. S.inf = infected S group, S.ctl = control S group, R.inf = infected R group, R.ctl = control R group. (PDF) [file pone.0045486.s005.pdf]

**A**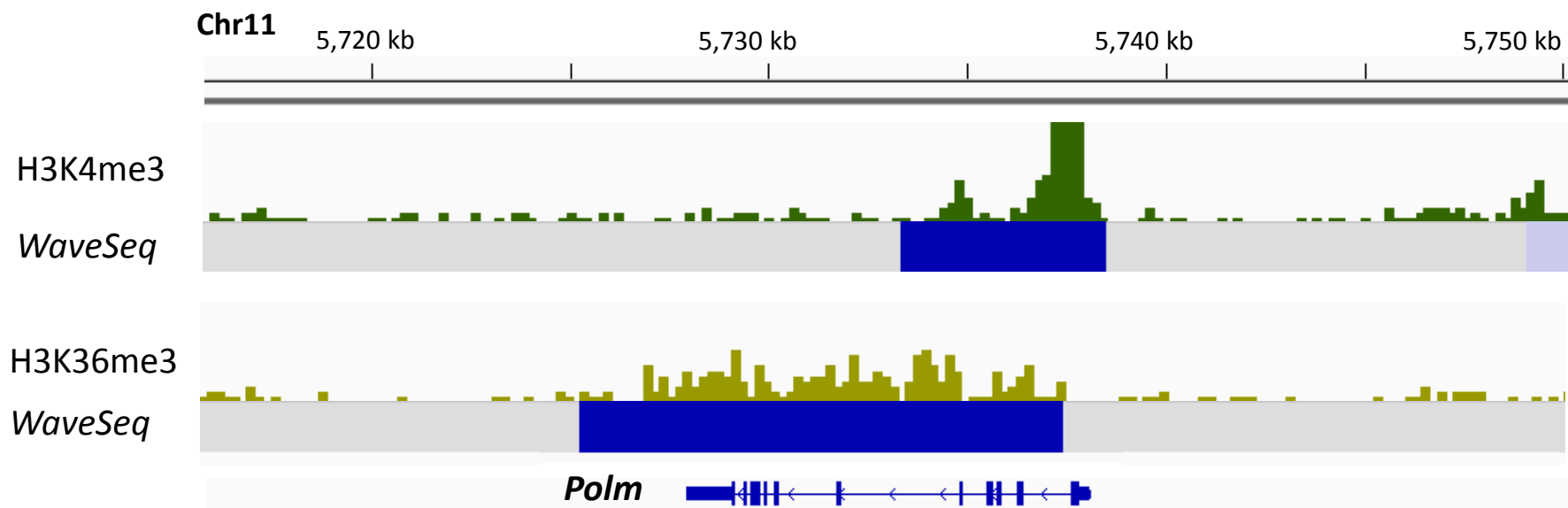**B**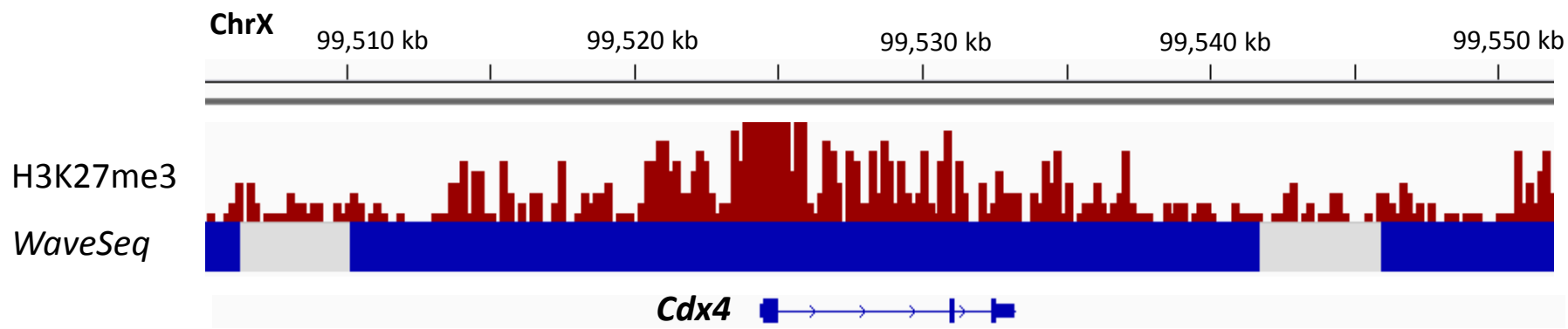

Supplement: Figure S6 — WaveSeq detects a broad variety of enrichment regions with high accuracy. Examples of WaveSeq peak calls on MEF histone modification data. (a) WaveSeq detects H3K4me3 and H3K36me3 marks on the housekeeping gene Polm located on chromosome 11 and (b) a broad peak of H3K27me3 on the developmental transcription factor Cdx4 which is silenced in differentiated cell populations. (PDF) [file pone.0045486.s006.pdf]
