# Supplementary material for: WaveSeq: A Novel Data-Driven Method of Detecting Histone Modification Enrichments Using Wavelets
Source: PLoS One. 2012 Sep 28;7(9):e45486. doi: 10.1371/journal.pone.0045486 (PMC3461018; doi:10.1371/journal.pone.0045486)
Supplement: Text S1 — Supplementary methods. Parameters used for published algorithms and data access information. (PDF) [file pone.0045486.s010.pdf]

Supplementary Information

WaveSeq: A Novel Data-driven Method of Detecting Histone Modification Enrichments using Wavelets

Apratim Mitra[1], Jiuzhou Song[1]

[1]Department of Animal and Avian Sciences, University of Maryland, College Park, MD 20742, USA

WaveSeq parameters

We carried out systematic optimization of the WaveSeq peak calling algorithm. We first assessed the effect of the number of samples (*N*) in the Monte Carlo threshold estimation step. The wavelet coefficient thresholds quickly reached saturation for all scales (Figure 1). Therefore, we chose $N = 5000$ for optimal accuracy and speed. The sampling was performed chromosome-by-chromosome.

There was marked variation in wavelet coefficient thresholds for different chromosomes at a specified p-value (Figure 2). There are two possible reasons for this: the number of enrichments on a specific chromosome and the chromosome size. The first arises out of the natural variation of different data sets and the latter out of the particular choice of the length and number of samples. In either case, this variation represents important information about the data and we account for it in our algorithm as follows: The mean and standard deviation of wavelet coefficient thresholds for each scale across the chromosomes were calculated and wavelet coefficients from the wavelet transform of the data were considered significant at the specified p-value if it was greater than the mean + standard deviation. The p-value for a significant wavelet power at a window was chosen to be $p = 0.2$ for punctate data sets (transcription factors and H3K4me3) and $p = 0.4$ for broad marks (H3K36me3 and H3K27me3).

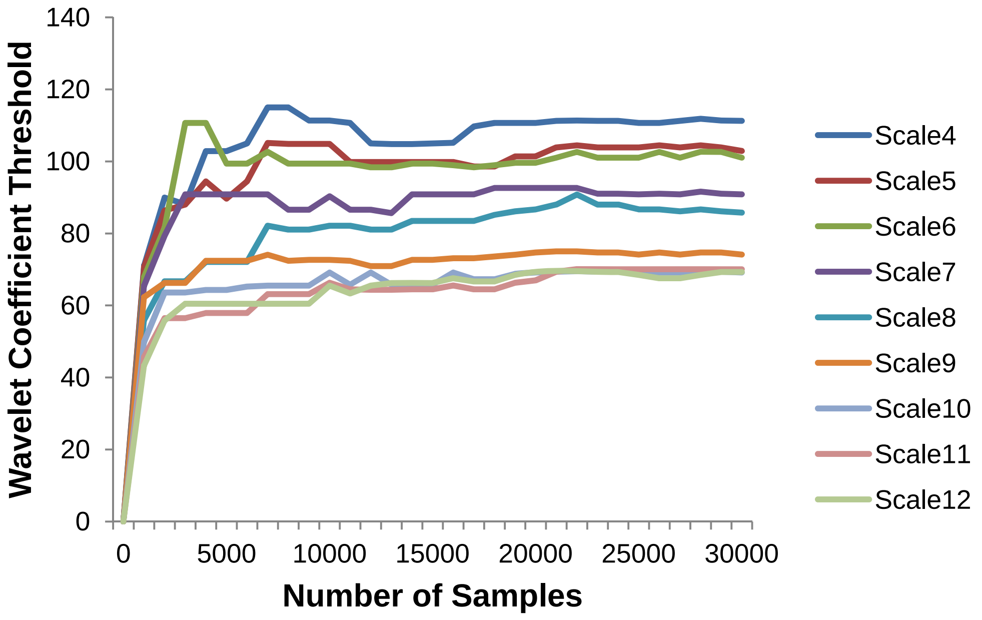

Figure 1: Wavelet coefficient thresholds reach saturation quickly.
Morlet wavelet thresholds at $p < 0.001$ of H3K4me3 data from chromosome 1 in the chicken bursal samples (S.inf group, window size = 200 bp).

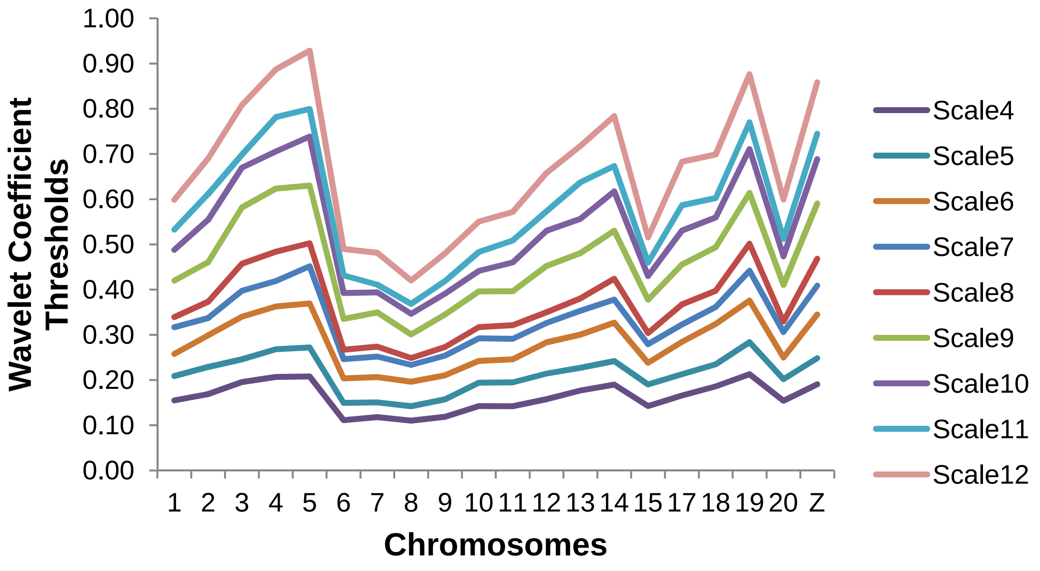

Figure 2: Comparison of wavelet coefficient thresholds for different chromosomes (p = 0.2). A variation is observed in the wavelet coefficient thresholds for different chromosomes of the chicken genome. Various factors may be responsible for this observation ranging from different chromosome lengths, the number of enrichment regions and the choices of size and number of samples. Bursa H3K4me3 data from the S.inf group was used for the above plot.

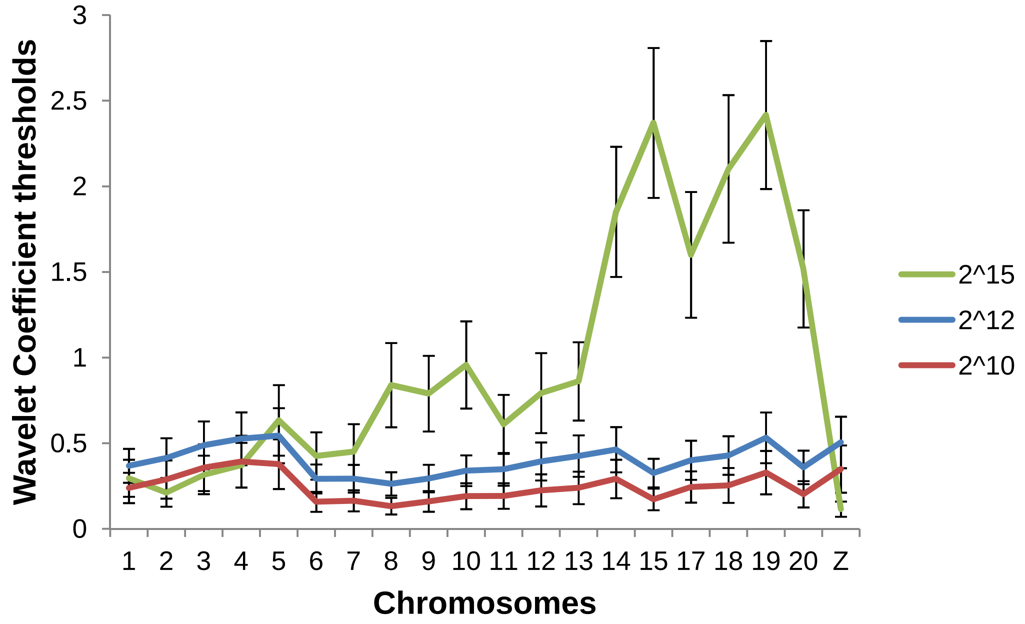

Figure 3: Effect of sample length on wavelet coefficient thresholds (p = 0.2). At higher sample sizes, wavelet coefficient thresholds are larger but the effect is only noticeable for the smaller chromosomes (13-20) of the chicken genome and is possibly due to oversampling. There is little difference between sample size of 2^{10} and 2^{12}. The error bars depict the standard errors over 9 scales (4-12) for sample lengths 2^{12} and 2^{15} and 7 scales (4-10) for sample length 2^{10}. The data corresponds to bursa H3K4me3 from the S.inf group.

Wavelet coefficient thresholds were larger for greater sample sizes but the effect was more pronounced for smaller chromosomes (Figure 3). This was possibly due to oversampling effects as the increase in wavelet coefficients was inversely correlated with chromosome size. We found a strong negative power law correlation between chromosome size and wavelet coefficient thresholds for sample length 2^{15} (R^2 = 0.7765, Figure 4) which was absent for smaller samples (2^{12}: R^2 = 0.0299; 2^{10}: R^2 = 0.1198). Greater sample lengths, therefore, are biased by chromosome size that could lead to large variations in coefficient thresholds. A smaller sample, on the other hand, could lead to lower wavelet coefficient thresholds and possibly more false positives. These two effects appeared to be reduced at a sample size of 2^{12} and hence we chose this for subsequent experiments. To further minimize the effect of chromosome size, we only considered chromosomes that have at least twice the length of the sample.

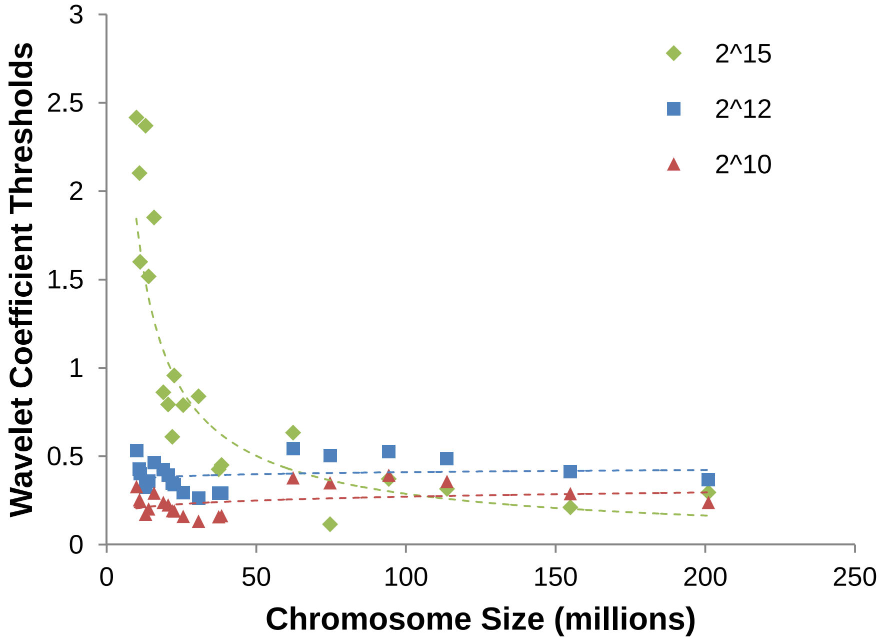

Figure 4: Correlation of chromosome size and wavelet coefficient thresholds.
Large sample lengths have a strong negative correlation (2^{15}: R^2 = 0.7765) with chromosome size which follows a power law distribution. This correlation is absent for smaller samples. The dotted lines represent power law regression lines for different sample lengths. The data corresponds to bursa H3K4me3 from the S.inf group.

The minimum scale considered for peak calling was $s = 4$, since lower scales are representative of broader patterns that are more likely to be background noise. We also noticed that a significant ChIP-Seq peak was significant at several scales simultaneously (See Figures 1B, C in main text) while localized peaks had fewer significant scales. Therefore, to further eliminate spurious peak calls due to local fluctuations, a window was considered significant only if there were at least 2 significant scales for the window.

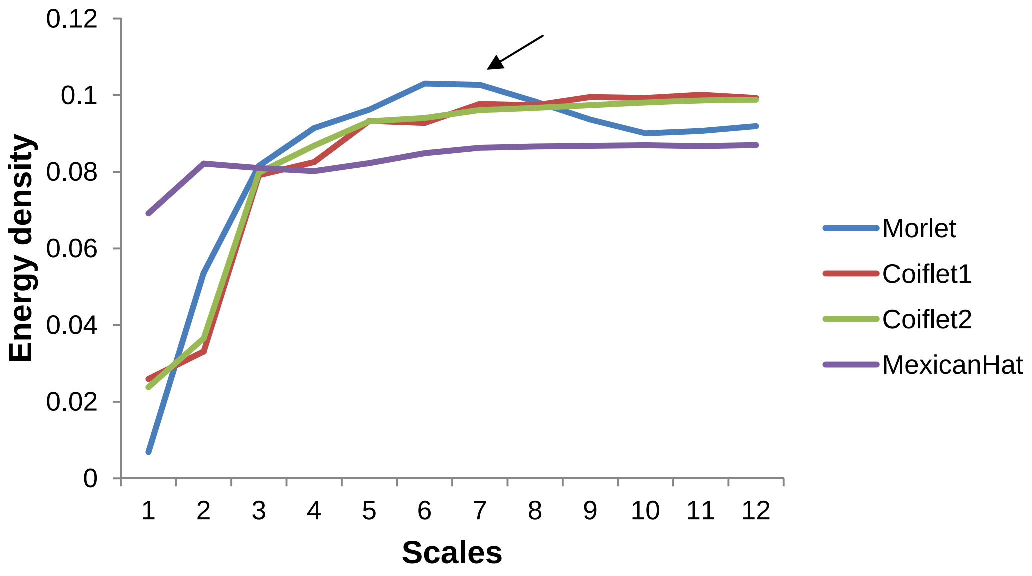

Figure 5: Comparison of wavelet energies for different wavelets.
A comparison of wavelet energies shows a higher density in a smaller band of scales for the Morlet wavelet as shown by the arrow-head. Other wavelets have more broadly distributed energy densities. Bursa H3K4me3 data from chromosome 2 of the S.inf group was used to obtain the above.

We also applied several different wavelet mother functions to ChIP-Seq data e.g. Morlet, Coiflets 1 and 2 and Mexican hat, to find the wavelets most suited to the data sets. All wavelets performed comparably when applied to punctate ChIP-Seq data sets but the morlet wavelet outperformed the others in detecting broad enrichment regions upto ~10kb while the Mexican hat wavelet was the most effective in calling very broad peaks (e.g. H3K27me3). Indeed a comparison of the energies at the various scales of the wavelet transform showed a higher density in a smaller band for the morlet wavelet and a more uniform distribution for the Mexican hat wavelet (Figure 4). The energy compression characteristic of the morlet wavelet represents a higher discriminative power over a smaller subset of scales and explains its performance for relatively strong enrichment patterns. The diffuse distribution of Mexican hat, however, is a better fit for the dispersed H3K27me3 marks as evidenced by its greater sensitivity for this dataset.

ChIP-Seq data sets

The GABP and NRSF data from human Jurkat cells were downloaded from:

- http://mendel.stanford.edu/SidowLab/downloads/quest/

H3K4me3, H3K36me3 and H3K27me3 data from murine embryonic fibroblasts were downloaded from:

- http://www.broadinstitute.org/scientific-community/science/programs/epigenomics/chip-seq-data

The list of qPCR validated sites for GABP and NRSF were obtained from [1]. The synthetic spike-in data were downloaded from:

- http://bioserver.hci.utah.edu/SupplementalPaperInfo/2008/Nix_EmpiricalMethods/

The “JohnsonSpikeDataHg17Low” data set was used for specificity benchmarks which was generated using human input control data from [2]. All data was downloaded in aligned format with read lengths of 25 bp for the GABP and NRSF data and approximately 32 bp for the H3K4me3 and H3K27me3 data.

Method parameters

We downloaded and configured the tested algorithms as follows:

1) FindPeaks v 4.0.15 was downloaded as part of the Vancouver Short Read Analysis Toolkit (VSRAT) from http://vancouvershortr.sourceforge.net. The reads in BED format were first separated into chromosomes using SeparateReads.jar. The following parameters were then used for FindPeaks.jar:

-aligner bed
-dist_type 0 190

2) MACS v 1.3.7.1 was downloaded from http://liulab.dfci.harvard.edu/MACS/. The following parameters were used:

--shiftsize=95
--nomodel True

For applying MACS to histone modification data sets, we used the additional parameter --nolambda as recommended by [3].

3) SiSSRs v 1.4 was downloaded from http://sissrs.rajajothi.com/. The following parameters were used:

-F 190

4) SICER v 1.1 was downloaded from http://home.gwu.edu/~wpeng/Software.htm. The following parameters were used:

Gap size = 2 (H3K4me3), 5 (H3K36me3) and 10 (H3K27me3)
E-value = 100

Window size = 200

5) RSEG was downloaded from http://smithlab.usc.edu/histone/rseg/. The following parameters were used:

-i 20

For the transcription factor binding site detection all methods were configured to have p-value < 0.001 in single sample experiments and p < 0.01 in the presence of matched controls. For uniformity, we set genome size = 3,107,000,000 bp for the GABP and NRSF (hg18) data sets and 2,725,000,000 bp for murine embryonic fibroblast (mm8) histone modification data. Recommended values were used for all other parameters.

References

1. Wilbanks EG, Facciotti MT (2010) Evaluation of algorithm performance in ChIP-seq peak detection. PLoS One 5: e11471.
2. Johnson DS, Mortazavi A, Myers RM, Wold B (2007) Genome-wide mapping of in vivo protein-DNA interactions. Science 316: 1497-1502.
3. Feng J, Liu T, Zhang Y (2011) Using MACS to Identify Peaks from ChIP-Seq Data. Current Protocols in Bioinformatics: John Wiley & Sons, Inc. pp. 2.14.11-12.14.14.
